# Central centrifugal cicatricial alopecia is associated with longer time to diagnosis among scarring alopecias

**DOI:** 10.1016/j.jdin.2025.11.021

**Published:** 2025-12-05

**Authors:** Thomas Issa, Alexandra M. Klomhaus, Chandra Kasakevich, Jean Pickford, Carolyn Goh

**Affiliations:** a Division of Dermatology, University of California, Los Angeles, Los Angeles, California; b The Department of Medicine Statistics Core, University of California, Los Angeles, Los Angeles, California; c Scarring Alopecia Foundation, Philadelphia, Pennsylvania

**Keywords:** access to care, central centrifugal cicatricial alopecia (CCCA), cicatricial alopecia, diagnostic delay, frontal fibrosing alopecia (FFA), hair disorders, hair loss, health disparities, lichen planopilaris (LPP), racial disparities, scalp health, scarring alopecia, TTD

*To the Editor:* Scarring alopecia (SA) comprises hair loss conditions characterized by permanent destruction of hair follicles.^1^ Prognosis depends on timely diagnosis and treatment. Goals are to prevent progression and alleviate symptoms, although no cure exists.^2^ This study explores associations between time from symptom onset to diagnosis and SA type, race, income, and insurance.

We performed a secondary analysis using data from the CAPAIR (Cicatricial Alopecia Patient Assessment & Impact Report) survey, distributed by the Scarring Alopecia Foundation, which collected responses from 1048 patients. Data included demographics (gender, race/ethnicity, income, and insurance) and clinical timelines (birth year, symptom onset, and diagnosis year).

Time to diagnosis (TTD) was calculated as year at diagnosis minus year of symptom onset. Descriptive statistics summarized patient characteristics. Univariate and multivariable negative binomial regression models examined associations between predictors and TTD, reported as incidence rate ratios (IRRs) and 95% confidence intervals.

After excluding 14 respondents due to inaccurate data, 1034 participants were included. Mean TTD was 3.54 ± 5.69 years. Demographics and TTD are summarized in Table I. Central centrifugal cicatricial alopecia (CCCA) was more prevalent among Black patients (195 [84.0%]), while frontal fibrosing alopecia (FFA)/lichen planopilaris were more common in White patients (670 [92.0%], χ^2^ [4, *N* = total] = 664.0, *P* < .0001).

**Table I:** Participants’ demographic data and TTD in years

Attribute	Count (%)	TTD (mean ± SD)
Total	1034 (100%)	3.54 ± 5.69
Gender		
Female	1007 (97.4%)	3.50 ± 5.66
Male	26 (2.5%)	5.04 ± 6.63
Nonbinary	1 (0.1%)	1.00 ± 0.00
Race		
White	750 (72.5%)	2.87 ± 5.10
Black or African American	233 (22.5%)	5.47 ± 6.67
American Indian or Alaska Native	1 (0.1%)	0.00 ± 0.00
Asian	11 (1.1%)	4.55 ± 7.29
Other	29 (2.8%)	3.52 ± 7.58
Don't know	2 (0.2%)	8.50 ± 10.61
Prefer not to answer	8 (0.8%)	1.50 ± 2.14
Income		
Less than $10,000	9 (0.9%)	2.78 ± 3.67
$10,000-$20,000	13 (1.3%)	4.00 ± 4.80
$20,000-$40,000	45 (4.4%)	4.22 ± 5.42
$40,000-$60,000	76 (7.4%)	4.62 ± 7.05
$60,000-$80,000	119 (11.5%)	3.10 ± 5.11

*SD*, Standard deviation; *TTD*, time to diagnosis.

Univariate analysis results are summarized in the forest plot in Fig 1. TTD was significantly longer in Black patients (5.47 ± 6.67 years) compared with White patients (2.87 ± 5.10 years) (IRR = 1.91, *P* < .0001) and in CCCA patients (5.67 ± 7.16 years) compared with FFA/lichen planopilaris (2.88 ± 5.04 years) (IRR = 1.97, *P* < .0001). Patients with income $150,000 to $250,000 and > $250,000 had shorter TTD compared with those earning < $60,000 (IRR = 0.729, *P* = .0397 and IRR = 0.548, *P* = .0004, respectively). In the multivariable model, only SA type remained significant, with CCCA showing significantly longer TTD (*P* = .0004).

**Fig 1:**
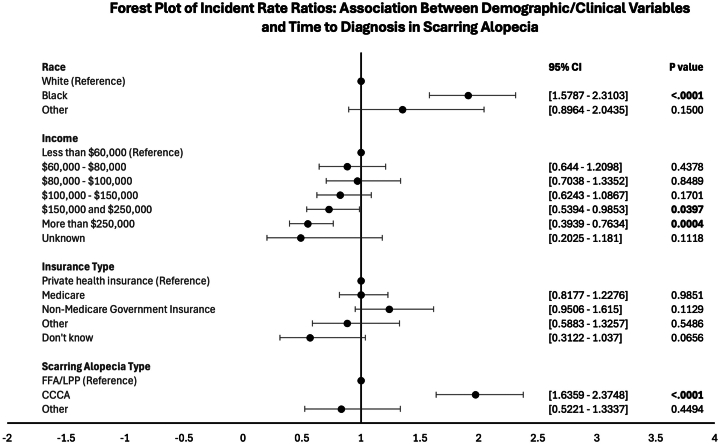
Forest plot of IRRs for TTD in SA by socioeconomic and clinical factors. *IRRs*, Incidence rate ratios; *SA*, scarring alopecia; *TTD*, time to diagnosis.

Although only SA type remained a significant predictor in multivariable analysis, interactions between multiple variables reduces statistical power; therefore, race and/or income may still be a factor in TTD. Furthermore, CCCA is thought to predominantly affect Black women;^3^ however, in our study, 14% of CCCA patients were White and had a very long TTD, potentially mitigating any potential effect of race.

Overall, these diagnostic delays may result from challenges in diagnosing CCCA, which can mimic traction alopecia and seborrheic dermatitis and have atypical presentations.^4^ Histopathologic overlap among lymphocytic SA further complicates diagnosis. Limited awareness among primary care providers as well as patients themselves may contribute. Additional factors may include distrust of healthcare and perceptions of inadequate understanding of Afro-textured hair.^5^ Diagnostic delays in SA can also relate to its earlier age of onset compared to FFA.

Recall bias is a limitation as the data were self-reported. Participants come from a patient advocacy group and have healthcare access, skewing toward higher income, insured, White individuals. Broader epidemiologic studies are needed.

Patients with SA are diagnosed several years after symptom onset. SA type (CCCA), not race, income, or insurance was the only independent predictor of longer TTD. Black race remains an important consideration due to its strong association with CCCA. Increased awareness and education among patients and providers may improve timely referrals to dermatology.

## Conflicts of interest

Dr Goh has served as a speaker for Pfizer, Inc. and Sun Pharmaceuticals and as a consultant for AbbVie, Arcutis, and Veradermics, receiving compensation for all roles. Thomas Issa, Dr Klomhaus, Chandra Kasakevich, and Jean Pickford have no conflicts of interest to declare.

## References

[1] Scarring (Cicatricial) alopecia: what it looks like & treatment. Cleveland clinic. https://my.clevelandclinic.org/health/diseases/24582-scarring-alopecia.

[2] Fechine C.O.C., Valente N.Y.S., Romiti R. (2022). Lichen planopilaris and frontal fibrosing alopecia: review and update of diagnostic and therapeutic features. Bras Dermatol.

[3] Balazic E., Chen A., Konisky H. (2023). A retrospective chart review of central centrifugal cicatricial alopecia patients at a single urban institution. JAAD Int.

[4] Sow Y.N., Jackson T.K., Taylor S.C., Ogunleye T.A. (2024). Lessons from a scoping review: clinical presentations of central centrifugal cicatricial alopecia. J Am Acad Dermatol.

[5] Gathers R.C., Mahan M.G. (2014). African American women, hair care, and health barriers. J Clin Aesthet Dermatol.

